# A neutral cubane with a Zn^II^_4_O_4_ core: tetra­benzoato­tetra­kis(μ_3_-hydroxydi-2-pyridylmethano­lato)tetra­zinc(II)–acetone–methanol (1/2/1)

**DOI:** 10.1107/S1600536809017772

**Published:** 2009-05-20

**Authors:** Dong Hoon Shin, Sim-Hee Han, Pan-Gi Kim, Cheal Kim, Youngmee Kim

**Affiliations:** a Department of Fine Chemistry, and Eco-Product and Materials Education Center, Seoul National University of Technology, Seoul 139-743, Republic of Korea; b Division of Forest Genetic Resources, Korea Forest Research Institute, Suwon, Gyeonggi-Do 441-350, Republic of Korea; c Department of Forest & Environment Resources, Kyungpook National University, Sangju 742-711, Republic of Korea; d Department of Chemistry and Nano Science, Ewha Womans University, Seoul 120-750, Republic of Korea

## Abstract

In the title compound, [Zn_4_(C_11_H_9_N_2_O_2_)_4_(C_7_H_5_O_2_)_4_]·2(CH_3_)_2_CO·CH_3_OH, the tetra­nuclear mol­ecule lies on a fourfold inversion axis. Zn^II^ ions and μ_3_-O atoms in the cubane core occupy alternating vertices, forming two inter­penetrating tetra­hedra. Each Zn^II^ ion is further coordinated by two N atoms from two different (py)_2_C(OH)O ligands (py is pyrid­yl) and one O atom from a monodentate benzoate ligand, forming a distorted octa­hedral environment. The (py)_2_C(OH)O ligand acts in an η^1^:η^3^:η^1^:μ_3_ manner, forming two five-membered ZnNCCO chelating rings with two different Zn^II^ atoms sharing a common C—O bond, and an alkoxide-type bond to a third Zn^II^ ion. There are four symmetry-related intra­molecular O—H⋯O hydrogen bonds between the two types of ligands. In the asymmetric unit, there is a half-occupancy acetone solvent mol­ecule and a half-occupancy methanol solvent molecule that lies on a twofold rotation axis.

## Related literature

For background to transition metal ions as the major cationic contributors to the inorganic composition of natural water and biological fluids, see: Daniele *et al.* (2008 ▶); For related crystal structures, see: Lee *et al.* (2008 ▶); Park *et al.* (2008 ▶); Yu *et al.* (2008 ▶); Stoumpos *et al.* (2008 ▶); Papaefstathiou & Perlepes (2002 ▶); Papatriantafyllopoulou *et al.* (2007 ▶).
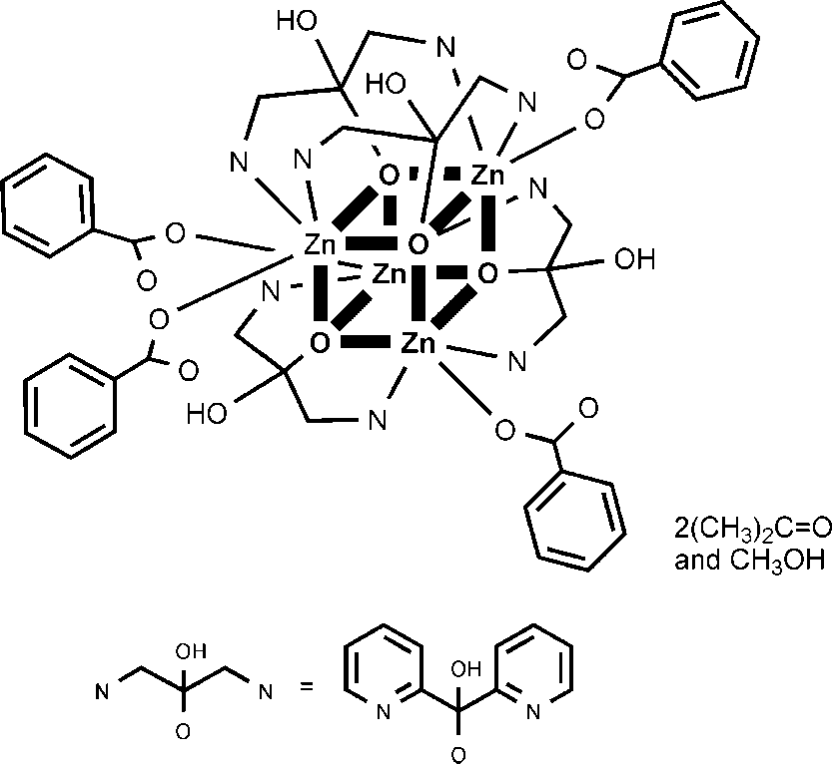


## Experimental

### 

#### Crystal data

[Zn_4_(C_11_H_9_N_2_O_2_)_4_(C_7_H_5_O_2_)_4_]·2C_3_H_6_O·CH_4_O
*M* *_r_* = 6795.70
Tetragonal, 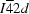

*a* = 14.3201 (4) Å
*c* = 37.730 (2) Å
*V* = 7737.1 (5) Å^3^
*Z* = 1
Mo *K*α radiation
μ = 1.30 mm^−1^
*T* = 170 K
0.10 × 0.08 × 0.05 mm

#### Data collection

Bruker SMART CCD diffractometer
Absorption correction: multi-scan (*SADABS*; Bruker, 1997 ▶) *T*_min_ = 0.883, *T*_max_ = 0.937
22787 measured reflections
4628 independent reflections
4279 reflections with *I* > 2σ(*I*)
*R*_int_ = 0.029

#### Refinement

*R*[*F* ^2^ > 2σ(*F* ^2^)] = 0.034
*wR*(*F* ^2^) = 0.110
*S* = 1.09
4628 reflections
245 parameters
5 restraints
H-atom parameters constrained
Δρ_max_ = 1.37 e Å^−3^
Δρ_min_ = −0.42 e Å^−3^
Absolute structure: Flack (1983 ▶), 2045 Friedel pairs
Flack parameter: 0.002 (13)

### 

Data collection: *SMART* (Bruker, 1997 ▶); cell refinement: *SAINT* (Bruker, 1997 ▶); data reduction: *SAINT*; program(s) used to solve structure: *SHELXS97* (Sheldrick, 2008 ▶); program(s) used to refine structure: *SHELXL97* (Sheldrick, 2008 ▶); molecular graphics: *SHELXTL* (Sheldrick, 2008 ▶); software used to prepare material for publication: *SHELXTL*.

## Supplementary Material

Crystal structure: contains datablocks I, global. DOI: 10.1107/S1600536809017772/lh2815sup1.cif

Structure factors: contains datablocks I. DOI: 10.1107/S1600536809017772/lh2815Isup2.hkl

Additional supplementary materials:  crystallographic information; 3D view; checkCIF report

## Figures and Tables

**Table 1:** Hydrogen-bond geometry (Å, °)

*D*—H⋯*A*	*D*—H	H⋯*A*	*D*⋯*A*	*D*—H⋯*A*
O2—H2*O*⋯O4	0.86	1.81	2.664 (3)	172

**Fig. 1.**
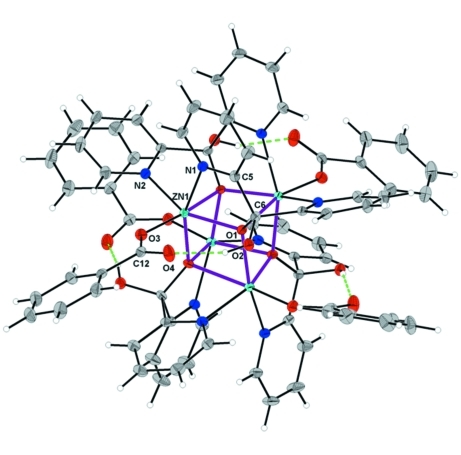
Partially labeled molecular structure of the title complex. Displacement ellipsoids areshown at the 30% probability level. The green dotted lines represent hydrogen bonds. Solvent molecules are not shown.

## supplementary crystallographic information

### Comment

Transition metal ions have, recently, received attention as the major cationic contributors to the inorganic composition of natural water and biological fluids (Daniele, *et al.*, 2008). While the main interest is focused on the interaction of transition metal ions with biologically active molecules such as amino acids, proteins, sugars and nucleotides, the study on the interaction of the transition metal ions with fulvic acids and humic acids, mainly found in soil, is in incipient stages. As models to examine the interaction, therefore, we have previously used copper(II) benzoate as a building block and reported the structures of copper(II) benzoates with quinoxaline, 6-methylquinoline, and 3-methylquinoline (Lee, *et al.*, 2008; Yu, *et al.*, 2008; Park, *et al.*, 2008). In this work, we have employed zinc(II) benzoate as a building block and di-2-pyridyl ketone as a ligand. We report herein the structure of the product of zinc(II) benzoate with di-2-pyridyl ketone.

The crystal structure contains tetranuclear [Zn_4_(O_2_CPh)_4_{(py)_2_C(OH)O}_4_] molecules (Fig. 1), similar to the corresponding Mn_4_ cubane compound (Stoumpos, *et al.*, 2008). The tetramolecular molecule lies on a fourfold inversion center and hence the asymmetric unit contains a quarter of a molecule. Zn^II^ ions and µ_3_-O atoms in the cubane [Zn^II^_4_(µ_3_-OR)_4_]^4+^ core occupy alternate vertices. Thus, the molecule consists of two interpenetrating tetrahedra: one contains four µ_3_-O atoms originating from the (py)_2_C(OH)O ligands, and the other contains four Zn^II^ atoms. Each Zn^II^ center is coordinated by two N atoms from two different (py)_2_C(OH)O ligands and one O atom from a monodentate PhCO_2_^-^ ligand to form a distorted octahedral geometry. The (py)_2_C(OH)O ligands acts as η^1^:η^3^:η^1^:µ_3_ to form two five-membered ZnNCCO chelating rings with two different Zn^II^ ions sharing a common C—O edge and an alkoxide-type bond to a third Zn^II^ ion. This ligation mode is common for the hydrated di-2-pyridyl ketone, (py)_2_C(OH)O^-^ (Papaefstathiou & Perlepes, 2002; Papatriantafyllopoulou,*et al.*, 2007). There are intramolecular hydrogen bonds interactions between the protonated O atom of the (py)_2_C(OH)O ligand and the uncoordinated O atom of the monodentate PhCO_2_ group.

### Experimental

38.0 mg (0.125 mmol) of Zn(NO_3_)_2_^.^6H_2_O and 35.5 mg (0.25 mmol) of C_6_H_5_COONH_4_ were dissolved in 4 ml water and carefully layered by 4 ml solution of a mixture of acetone, methanol and ethanol (2/2/2) of di-2-pyridyl ketone ligand (46.1 mg, 0.25 mmol). Crystals of the title compound suitable for X-ray analysis were obtained in a few weeks.

### Refinement

H atoms were placed in calculated positions with C—H distances of 0.93–0.98 Å and O—H = 0.82 Å. They were included in the refinement in a riding-motion approximation with *U*_iso_(H) = 1.2*U*_eq_(C) or 1.5*U*_eq_(C_methyl_ and O).

### Crystal data

**Table:**

[Zn_4_(C_11_H_9_N_2_O_2_)_4_(C_7_H_5_O_2_)_4_]·2C_3_H_6_O·CH_4_O	*D*_x_ = 1.458 Mg m^−^^3^
*M**_r_* = 6795.70	Mo *K*α radiation, λ = 0.71073 Å
Tetragonal, *I*42*d*	Cell parameters from 8208 reflections
Hall symbol: I -4 2bw	θ = 2.8–27.0°
*a* = 14.3201 (4) Å	µ = 1.30 mm^−^^1^
*c* = 37.730 (2) Å	*T* = 170 K
*V* = 7737.1 (5) Å^3^	Polyhedron, colorless
*Z* = 1	0.10 × 0.08 × 0.05 mm
*F*(000) = 3496	

### Data collection

**Table:**

Bruker SMART CCD diffractometer	4628 independent reflections
Radiation source: fine-focus sealed tube	4279 reflections with *I* > 2σ(*I*)
graphite	*R*_int_ = 0.029
φ and ω scans	θ_max_ = 28.3°, θ_min_ = 1.5°
Absorption correction: multi-scan (*SADABS*; Bruker, 1997)	*h* = −18→18
*T*_min_ = 0.883, *T*_max_ = 0.937	*k* = −9→18
22787 measured reflections	*l* = −48→48

### Refinement

**Table:**

Refinement on *F*^2^	Secondary atom site location: difference Fourier map
Least-squares matrix: full	Hydrogen site location: inferred from neighbouring sites
*R*[*F*^2^ > 2σ(*F*^2^)] = 0.034	H-atom parameters constrained
*wR*(*F*^2^) = 0.110	*w* = 1/[σ^2^(*F*_o_^2^) + (0.0787*P*)^2^ + 0.4409*P*] where *P* = (*F*_o_^2^ + 2*F*_c_^2^)/3
*S* = 1.09	(Δ/σ)_max_ = 0.001
4628 reflections	Δρ_max_ = 1.37 e Å^−^^3^
245 parameters	Δρ_min_ = −0.42 e Å^−^^3^
5 restraints	Absolute structure: Flack (1983), 2045 Friedel pairs
Primary atom site location: structure-invariant direct methods	Flack parameter: 0.002 (13)

### Special details

**Table:**

Geometry. All e.s.d.'s (except the e.s.d. in the dihedral angle between two l.s. planes) are estimated using the full covariance matrix. The cell e.s.d.'s are taken into account individually in the estimation of e.s.d.'s in distances, angles and torsion angles; correlations between e.s.d.'s in cell parameters are only used when they are defined by crystal symmetry. An approximate (isotropic) treatment of cell e.s.d.'s is used for estimating e.s.d.'s involving l.s. planes.
Refinement. Refinement of *F*^2^ against ALL reflections. The weighted *R*-factor *wR* and goodness of fit *S* are based on *F*^2^, conventional *R*-factors *R* are based on *F*, with *F* set to zero for negative *F*^2^. The threshold expression of *F*^2^ > σ(*F*^2^) is used only for calculating *R*-factors(gt) *etc*. and is not relevant to the choice of reflections for refinement. *R*-factors based on *F*^2^ are statistically about twice as large as those based on *F*, and *R*- factors based on ALL data will be even larger.

### Fractional atomic coordinates and isotropic or equivalent isotropic displacement parameters (Å^2^)

**Table:**

	*x*	*y*	*z*	*U*_iso_*/*U*_eq_	Occ. (<1)
Zn1	0.61621 (2)	0.51226 (2)	0.470190 (8)	0.01677 (10)	
O1	0.50301 (15)	0.59983 (13)	0.47540 (5)	0.0177 (4)	
O2	0.53176 (14)	0.75354 (15)	0.45808 (6)	0.0237 (5)	
H2O	0.5908	0.7429	0.4575	0.036*	
O3	0.73924 (15)	0.58089 (15)	0.47172 (6)	0.0257 (4)	
O4	0.71640 (16)	0.73233 (17)	0.46138 (9)	0.0390 (6)	
N1	0.58789 (18)	0.55925 (18)	0.41603 (6)	0.0197 (5)	
N2	0.67260 (17)	0.38313 (19)	0.45377 (6)	0.0204 (5)	
C1	0.6300 (2)	0.5287 (2)	0.38641 (8)	0.0262 (6)	
H1	0.6712	0.4768	0.3881	0.031*	
C2	0.6162 (3)	0.5691 (3)	0.35381 (9)	0.0366 (8)	
H2	0.6465	0.5455	0.3333	0.044*	
C3	0.5566 (3)	0.6455 (3)	0.35178 (10)	0.0404 (10)	
H3	0.5448	0.6748	0.3296	0.049*	
C4	0.5141 (3)	0.6789 (3)	0.38253 (8)	0.0331 (8)	
H4	0.4741	0.7318	0.3818	0.040*	
C5	0.5320 (2)	0.6328 (2)	0.41431 (8)	0.0210 (6)	
C6	0.4901 (2)	0.6676 (2)	0.44992 (7)	0.0187 (5)	
C7	0.6154 (2)	0.3128 (2)	0.44535 (7)	0.0192 (5)	
C8	0.6484 (2)	0.2278 (2)	0.43286 (9)	0.0256 (6)	
H8	0.6064	0.1783	0.4277	0.031*	
C9	0.7445 (3)	0.2165 (2)	0.42796 (10)	0.0325 (8)	
H9	0.7685	0.1604	0.4181	0.039*	
C10	0.8032 (2)	0.2871 (3)	0.43751 (10)	0.0330 (8)	
H10	0.8689	0.2797	0.4353	0.040*	
C11	0.7658 (2)	0.3707 (2)	0.45060 (9)	0.0280 (7)	
H11	0.8069	0.4197	0.4574	0.034*	
C12	0.7640 (2)	0.6651 (2)	0.47105 (9)	0.0227 (6)	
C13	0.8626 (2)	0.6836 (2)	0.48381 (8)	0.0237 (6)	
C14	0.9023 (2)	0.7726 (2)	0.48046 (11)	0.0341 (8)	
H14	0.8676	0.8218	0.4699	0.041*	
C15	0.9924 (3)	0.7888 (3)	0.49264 (12)	0.0461 (10)	
H15	1.0196	0.8489	0.4900	0.055*	
C16	1.0423 (3)	0.7181 (3)	0.50846 (13)	0.0474 (11)	
H16	1.1039	0.7295	0.5168	0.057*	
C17	1.0032 (3)	0.6313 (3)	0.51216 (11)	0.0398 (8)	
H17	1.0374	0.5829	0.5234	0.048*	
C18	0.9141 (2)	0.6138 (3)	0.49960 (9)	0.0306 (7)	
H18	0.8882	0.5530	0.5019	0.037*	
O1S	0.9292 (16)	1.1086 (15)	0.3972 (6)	0.218 (6)*	0.50
C1S	0.9466 (13)	1.0280 (17)	0.3923 (6)	0.218 (6)*	0.50
C2S	0.965 (2)	0.972 (2)	0.4275 (8)	0.218 (6)*	0.50
H2S1	1.0259	0.9886	0.4372	0.328*	0.50
H2S2	0.9634	0.9045	0.4224	0.328*	0.50
H2S3	0.9160	0.9865	0.4448	0.328*	0.50
C3S	0.9503 (19)	0.975 (2)	0.3559 (7)	0.218 (6)*	0.50
H3S1	0.8956	0.9340	0.3537	0.328*	0.50
H3S2	1.0073	0.9370	0.3547	0.328*	0.50
H3S3	0.9504	1.0201	0.3364	0.328*	0.50
O2S	0.7500	1.0096 (11)	0.3750	0.110 (5)*	0.50
H2S	0.7197	1.0292	0.3574	0.164*	0.25
C21S	0.7500	0.9049 (11)	0.3750	0.139 (10)*	0.50
H21A	0.7999	0.8821	0.3595	0.208*	0.25
H21B	0.6896	0.8821	0.3664	0.208*	0.25
H21C	0.7605	0.8821	0.3992	0.208*	0.25

### Atomic displacement parameters (Å^2^)

**Table:**

	*U*^11^	*U*^22^	*U*^33^	*U*^12^	*U*^13^	*U*^23^
Zn1	0.01658 (17)	0.01739 (17)	0.01634 (15)	0.00064 (12)	0.00086 (12)	0.00068 (12)
O1	0.0189 (9)	0.0175 (9)	0.0167 (9)	0.0029 (8)	0.0001 (8)	0.0034 (7)
O2	0.0206 (10)	0.0193 (10)	0.0311 (11)	−0.0009 (9)	0.0009 (8)	−0.0005 (9)
O3	0.0216 (11)	0.0258 (11)	0.0296 (11)	−0.0035 (9)	0.0013 (9)	−0.0002 (10)
O4	0.0214 (11)	0.0262 (13)	0.0693 (19)	−0.0015 (9)	−0.0062 (11)	0.0019 (12)
N1	0.0201 (12)	0.0213 (12)	0.0176 (11)	0.0003 (9)	0.0009 (9)	0.0007 (9)
N2	0.0182 (12)	0.0232 (12)	0.0199 (11)	0.0025 (11)	0.0015 (9)	0.0025 (10)
C1	0.0244 (15)	0.0299 (16)	0.0244 (14)	0.0035 (13)	0.0059 (12)	0.0000 (12)
C2	0.044 (2)	0.044 (2)	0.0218 (15)	0.0112 (18)	0.0101 (15)	0.0016 (14)
C3	0.050 (2)	0.048 (2)	0.0227 (16)	0.0158 (19)	0.0113 (16)	0.0114 (15)
C4	0.0378 (19)	0.0376 (18)	0.0238 (15)	0.0120 (16)	0.0058 (14)	0.0085 (13)
C5	0.0206 (14)	0.0214 (14)	0.0211 (14)	0.0008 (12)	0.0015 (10)	0.0043 (11)
C6	0.0199 (14)	0.0168 (13)	0.0194 (12)	0.0026 (12)	0.0006 (11)	0.0040 (10)
C7	0.0206 (14)	0.0207 (13)	0.0163 (12)	0.0010 (12)	0.0005 (11)	0.0006 (10)
C8	0.0267 (15)	0.0255 (16)	0.0245 (15)	0.0056 (13)	−0.0002 (12)	−0.0017 (12)
C9	0.0317 (18)	0.0298 (17)	0.0361 (18)	0.0124 (15)	0.0060 (15)	−0.0024 (14)
C10	0.0231 (16)	0.0371 (19)	0.0388 (19)	0.0086 (14)	0.0068 (14)	0.0018 (15)
C11	0.0214 (15)	0.0312 (17)	0.0315 (17)	−0.0005 (13)	0.0033 (12)	0.0037 (14)
C12	0.0192 (14)	0.0267 (15)	0.0221 (14)	−0.0017 (11)	0.0049 (12)	−0.0030 (13)
C13	0.0204 (15)	0.0263 (16)	0.0246 (14)	−0.0009 (12)	0.0040 (12)	−0.0069 (12)
C14	0.0275 (18)	0.0260 (17)	0.049 (2)	−0.0028 (14)	0.0040 (14)	−0.0056 (14)
C15	0.0283 (18)	0.0291 (18)	0.081 (3)	−0.0061 (16)	−0.001 (2)	−0.0103 (19)
C16	0.0218 (17)	0.048 (2)	0.072 (3)	−0.0035 (16)	−0.0083 (18)	−0.020 (2)
C17	0.0292 (17)	0.038 (2)	0.052 (2)	0.0050 (16)	−0.0075 (17)	−0.0062 (17)
C18	0.0270 (16)	0.0290 (16)	0.0357 (17)	−0.0039 (14)	−0.0012 (13)	−0.0028 (14)

### Geometric parameters (Å, °)

**Table:**

Zn1—O3	2.018 (2)	C9—C10	1.365 (5)
Zn1—O1	2.059 (2)	C9—H9	0.9500
Zn1—O1^i^	2.0776 (19)	C10—C11	1.401 (5)
Zn1—N2	2.111 (3)	C10—H10	0.9500
Zn1—N1	2.189 (2)	C11—H11	0.9500
Zn1—O1^ii^	2.351 (2)	C12—C13	1.515 (4)
O1—C6	1.378 (3)	C13—C18	1.378 (5)
O1—Zn1^iii^	2.0777 (19)	C13—C14	1.401 (5)
O1—Zn1^ii^	2.352 (2)	C14—C15	1.389 (5)
O2—C6	1.402 (4)	C14—H14	0.9500
O2—H2O	0.8587	C15—C16	1.376 (6)
O3—C12	1.257 (4)	C15—H15	0.9500
O4—C12	1.235 (4)	C16—C17	1.371 (6)
N1—C5	1.325 (4)	C16—H16	0.9500
N1—C1	1.343 (4)	C17—C18	1.384 (5)
N2—C7	1.336 (4)	C17—H17	0.9500
N2—C11	1.352 (4)	C18—H18	0.9500
C1—C2	1.374 (5)	O1S—C1S	1.195 (10)
C1—H1	0.9500	C1S—C2S	1.57 (2)
C2—C3	1.391 (5)	C1S—C3S	1.57 (2)
C2—H2	0.9500	C2S—H2S1	0.9800
C3—C4	1.394 (5)	C2S—H2S2	0.9800
C3—H3	0.9500	C2S—H2S3	0.9800
C4—C5	1.392 (4)	C3S—H3S1	0.9800
C4—H4	0.9500	C3S—H3S2	0.9800
C5—C6	1.553 (4)	C3S—H3S3	0.9800
C6—C7^ii^	1.546 (4)	O2S—C21S	1.499 (2)
C7—C8	1.388 (4)	O2S—H2S	0.8400
C7—C6^ii^	1.546 (4)	C21S—H21A	0.98000
C8—C9	1.398 (5)	C21S—H21B	0.98000
C8—H8	0.9500	C21S—H21C	0.98000
			
O3—Zn1—O1	112.83 (9)	C7—C8—H8	120.6
O3—Zn1—O1^i^	96.98 (9)	C9—C8—H8	120.6
O1—Zn1—O1^i^	83.16 (8)	C10—C9—C8	119.1 (3)
O3—Zn1—N2	95.80 (9)	C10—C9—H9	120.5
O1—Zn1—N2	149.64 (9)	C8—C9—H9	120.5
O1^i^—Zn1—N2	103.94 (9)	C9—C10—C11	119.4 (3)
O3—Zn1—N1	92.22 (9)	C9—C10—H10	120.3
O1—Zn1—N1	75.88 (9)	C11—C10—H10	120.3
O1^i^—Zn1—N1	159.01 (9)	N2—C11—C10	121.4 (3)
N2—Zn1—N1	93.79 (10)	N2—C11—H11	119.3
O3—Zn1—O1^ii^	164.53 (8)	C10—C11—H11	119.3
O1—Zn1—O1^ii^	80.57 (8)	O4—C12—O3	126.7 (3)
O1^i^—Zn1—O1^ii^	76.33 (8)	O4—C12—C13	118.1 (3)
N2—Zn1—O1^ii^	72.80 (8)	O3—C12—C13	115.2 (3)
N1—Zn1—O1^ii^	98.82 (8)	C18—C13—C14	118.8 (3)
C6—O1—Zn1	117.87 (17)	C18—C13—C12	120.6 (3)
C6—O1—Zn1^iii^	126.55 (17)	C14—C13—C12	120.6 (3)
Zn1—O1—Zn1^iii^	104.24 (9)	C15—C14—C13	119.9 (4)
C6—O1—Zn1^ii^	108.97 (17)	C15—C14—H14	120.0
Zn1—O1—Zn1^ii^	98.51 (8)	C13—C14—H14	120.1
Zn1^iii^—O1—Zn1^ii^	94.78 (7)	C16—C15—C14	120.2 (4)
C6—O2—H2O	104.9	C16—C15—H15	119.9
C12—O3—Zn1	135.5 (2)	C14—C15—H15	119.9
C5—N1—C1	119.3 (3)	C17—C16—C15	119.9 (4)
C5—N1—Zn1	113.71 (19)	C17—C16—H16	120.0
C1—N1—Zn1	126.4 (2)	C15—C16—H16	120.0
C7—N2—C11	119.1 (3)	C16—C17—C18	120.5 (4)
C7—N2—Zn1	119.66 (19)	C16—C17—H17	119.8
C11—N2—Zn1	121.3 (2)	C18—C17—H17	119.8
N1—C1—C2	122.9 (3)	C13—C18—C17	120.6 (4)
N1—C1—H1	118.6	C13—C18—H18	119.7
C2—C1—H1	118.5	C17—C18—H18	119.7
C1—C2—C3	118.0 (3)	O1S—C1S—C2S	114 (2)
C1—C2—H2	121.0	O1S—C1S—C3S	128 (2)
C3—C2—H2	121.0	C2S—C1S—C3S	119 (2)
C2—C3—C4	119.4 (3)	C1S—C2S—H2S1	109.5
C2—C3—H3	120.3	C1S—C2S—H2S2	109.4
C4—C3—H3	120.3	H2S1—C2S—H2S2	109.5
C5—C4—C3	118.3 (3)	C1S—C2S—H2S3	109.5
C5—C4—H4	120.9	H2S1—C2S—H2S3	109.5
C3—C4—H4	120.9	H2S2—C2S—H2S3	109.5
N1—C5—C4	122.0 (3)	C1S—C3S—H3S1	109.5
N1—C5—C6	116.5 (2)	C1S—C3S—H3S2	109.4
C4—C5—C6	121.4 (3)	H3S1—C3S—H3S2	109.5
O1—C6—O2	114.1 (2)	C1S—C3S—H3S3	109.5
O1—C6—C7^ii^	109.6 (2)	H3S1—C3S—H3S3	109.5
O2—C6—C7^ii^	106.3 (2)	H3S2—C3S—H3S3	109.5
O1—C6—C5	109.0 (2)	C21S—O2S—H2S	109.5
O2—C6—C5	107.9 (2)	O2S—C21S—H21A	109.00
C7^ii^—C6—C5	109.8 (2)	O2S—C21S—H21B	109.00
N2—C7—C8	122.1 (3)	H21A—C21S—H21B	110.00
N2—C7—C6^ii^	115.9 (2)	O2S—C21S—H21C	109.00
C8—C7—C6^ii^	122.0 (3)	H21A—C21S—H21C	109.00
C7—C8—C9	118.8 (3)	H21B—C21S—H21C	109.00

Symmetry codes: (i) *y*, −*x*+1, −*z*+1; (ii) −*x*+1, −*y*+1, *z*; (iii) −*y*+1, *x*, −*z*+1.

### Hydrogen-bond geometry (Å, °)

**Table:**

*D*—H···*A*	*D*—H	H···*A*	*D*···*A*	*D*—H···*A*
O2—H2O···O4	0.86	1.81	2.664 (3)	172
